# Supplementary material for: SiO2 Fibers of Two Lengths and Their Effect on Cellular Responses of Macrophage-like Cells
Source: Molecules. 2022 Jul 12;27(14):4456. doi: 10.3390/molecules27144456 (PMC9320682; doi:10.3390/molecules27144456)
Supplement: Supplementary file 1 [file molecules-27-04456-s001.zip › molecules-1776486-supplementary.pdf]

molecules

MDPI

SiO_2 Fibers of Two Lengths and Their Effect on Cellular Responses of Macrophage-Like Cells

Denisa Smela [1,2,*], Chia-Jung Chang [2], Ludek Hromadko [3,4], Jan Macak [3,4], Zuzana Bilkova [1], Akiyoshi Taniguchi [2]

[1] Department of Biological and Biochemical Sciences, Faculty of Chemical Technology, University of Pardubice, Studentska 573, 532 10 Pardubice, Czech Republic
[2] Research Center for Functional Materials, National Institute for Materials Science, 1-1 Namiki, Tsukuba, Ibaraki 305-0044, Japan
[3] Center of Materials and Nanotechnologies, Faculty of Chemical Technology, University of Pardubice, Nam. Cs. Legii 565, 53002 Pardubice, Czech Republic
[4] Central European Institute of Technology, Brno University of Technology, Zerotinovo nam. 617/9, 601 77 Brno, Czech Republic
* Correspondence: Denisa.Smela@upce.cz

Figure S1 shows the difference in CD14 expression on the surface of THP-1 cells before and after differentiation with phorbol-12-myristate-13-acetate (PMA). Cells were treated with 50 ng/mL PMA in RPMI-1640 medium for 48 hours, then washed to discard remaining non-differentiated cells, refeeded with fresh medium without PMA, and left 24 hours to recover. After the differentiation, there is a clear increase in the signal intensity of immunostained CD14 in the second row of Figure S1 in comparison to the first row (non-differentiated control cells). There is also a change in cell morphology after the differentiation process. CD14 expression is further increased after treatment with 1 µg/mL LPS.
CD14 immunostaining: Cells were seeded at 4×10^4 per compartment in a CELLview™ dish with a glass bottom in medium with PMA. THP-1 monocytes in two compartments were differentiated. Afterwards, cells were washed with PBS, fixed for 20 min and permeabilized with 0.05% saponin for 10 min. Cells were washed and 3% bovine serum albumin (BSA) was added for blocking (1 hour). 1:200 Alexa Fluor® 488 Mouse Anti-Human CD14 (BD Pharmingen™, Allschwil, Switzerland) were added after blocking, followed by overnight incubation at 4 °C. Cells were washed and the nucleus was stained with 1:300 DAPI (Abcam, Cambridge, United Kingdom) for 15 min. Cells were incubated at room temperature (RT) and then washed again.

After staining of the target molecules, the images were acquired with a Zeiss LSM 510 META confocal microscope system (Carl Zeiss, Jena, Germany). The image analysis to measure the fluorescence intensity was performed by ImageJ software.

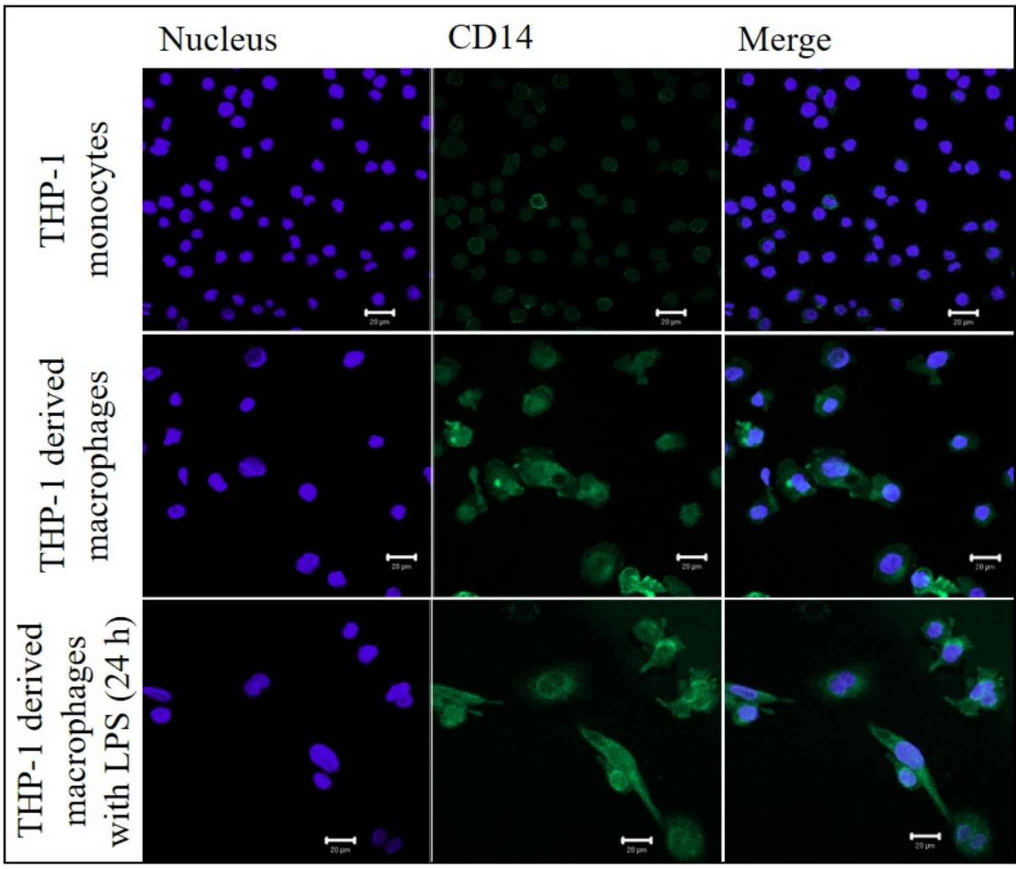

Figure S1: THP-1 cells before and after differentiation into macrophages were immunostained with Alexa Fluor® 488 Mouse Anti-Human CD14 (CD14 – green) and nucleus was stained with DAPI (blue). First row: THP-1 monocytes, second row: THP-1 derived macrophages, third row: THP-1 derived macrophages after treatment with LPS (1 μg/mL). Scale bar is 20 μm.
